# Indoleamine 2,3-dioxygenase-1 expression in non-small-cell lung cancer: analyses of prevalence, clinical correlations and prognostic impact in 2 large patient cohorts

**DOI:** 10.3389/fimmu.2025.1586782

**Published:** 2025-05-22

**Authors:** Francesco Agustoni, Hui Yu, Kim Ellison, Derek Smith, Paul Mitchell, Gareth Rivalland, Rafal Dziadziuszko, Dexiang Gao, Shengxiang Ren, Christopher J. Rivard, Inigo San Millan, Salvatore Corallo, Paolo Pedrazzoli, Fred R. Hirsch

**Affiliations:** ^1^ Department of Internal Medicine and Medical Therapeutics, University of Pavia, Pavia, Italy; ^2^ Medical Oncology Unit, Fondazione IRCCS Policlinico San Matteo, Pavia, Italy; ^3^ Division of Medical Oncology, School of Medicine, University of Colorado, Aurora, CO, United States; ^4^ School of Medicine, University of Colorado Cancer Center Biostatistics Shared Resources and Department of Pediatrics, Aurora, CO, United States; ^5^ Department of Medical Oncology, Austin Health, Olivia Newton – John Cancer and Wellness Centre, Melbourne, VIC, Australia; ^6^ Department of Oncology and Radiotherapy, Medical University, Gdansk, Poland; ^7^ Department of Medical Oncology, Shanghai Pulmonary Hospital and Thoracic Cancer Institute, Tongji University School of Medicine, Shanghai, China; ^8^ Division of Endocrinology, Metabolism and Diabetes, School of Medicine, University of Colorado, Aurora, CO, United States; ^9^ Department of Human Physiology and Nutrition, University of Colorado, Colorado Springs, CO, United States; ^10^ Center for Thoracic Oncology, Tisch Cancer Institute, Mount Sinai Health System, New York, NY, United States

**Keywords:** non-small cell lung cancer, indoleamine 2, 3-dioxygenase-1, immunotherapy, immunohistochemistry, biomarker

## Abstract

**Background:**

Indoleamine 2,3-dioxygenase-1 (IDO-1) is a cytosolic enzyme involved in the catabolism of tryptophan. IDO-1-related immune suppression is due to decreased tryptophan availability and to the generation of tryptophan metabolites, culminating in substantial suppression of T-lymphocytes. Here we investigate IDO-1 expression in 2 cohorts of non-small-cell lung cancer (NSCLC) specimens, both in tumor cells and in immune infiltrate, with correlation of IDO-1 to PD-L1 expression, clinical patient demographics and outcomes.

**Methods:**

Evaluation first utilized an exploratory cohort of 259 NSCLC samples obtained from 122 patients followed by a second validating cohort of 1,200 NSCLC samples obtained from 437 patients. All tumor samples were collected from patients who underwent surgical lung resections. IDO-1 expression was evaluated by immunohistochemistry (IHC). Correlations were assessed using Spearman and Kendall tests. A Cox proportional hazards (PH) model was used to assess if overall survival (OS) was associated with IDO-1 positivity in univariate and multivariable settings.

**Results:**

In the validating cohort of 437 patients IDO-1 expression was positive in 111 (25.4%) with an H-Score ≥ 1. IDO-1 expression was determined to be greater in tumor immune infiltrate, with 406 patients (93.8%) determined as positive. Both continuous and binary versions of tumor H-Score showed a significant positive correlation with the amount of tumor immune infiltrate (0.1806 and 0.1698, p < 0.0001). None of the analyzed variables (age, sex, histology, stage, EGFR, KRAS and PD-L1 status) were found to display a significant correlation with IDO-1 positivity in tumor and immune cells. IDO-1 positivity in tumor cells was found to be significantly associated with OS in the univariate setting and in the multivariable model [P-value = 0.009 and 0.021, respectively; HR: 0.72 (95% CI: 0.55-0.95)]. IDO-1 positivity in immune cells was found to be significantly associated with OS in the univariate setting and was borderline significant in the multivariable model [P-value = 0.006 and 0.053; HR: 0.798 (95% CI: 0.635-1.003)].

**Conclusion:**

Our results suggest a prognostic role of IDO-1 protein expression in NSCLC tumor and immune cells independent of EGFR, KRAS AND PD-L1 expression, and should be explored as a predictive biomarker in clinical studies with IDO-1 targeted therapies.

## Introduction

Lung cancer remains the leading cause of cancer mortality worldwide and non-small-cell lung cancer (NSCLC) accounts for more than 85% of all lung cancer (1, 2). For patients with advanced NSCLC (Stage IIIB-IV) the cornerstone first-line treatment until a few years ago was represented by a platinum-based doublet chemotherapy (CT) containing a third generation anticancer drug (3). Current alternative treatment options are based on the presence of genetic aberrations, such as sensitizing mutations of epidermal growth factor receptor (EGFR), translocations of anaplastic lymphoma kinase (ALK), or other molecular drivers potentially targeted by biological treatments in first-line (ROS1, BRAF, NTRK), or in second and later lines (MET, RET, KRAS). Most patients with NSCLC don’t harbor these oncogenic drivers and treatment options are limited to immunotherapy alone or in combination with CT. Immunotherapy has recently emerged as a promising therapeutic strategy for NSCLC, including adoptive T-cell transfer, dendritic cell vaccines, peptide vaccines, oncolytic viruses, cytokine therapy, agonist and antagonist monoclonal antibodies. Multiple clinical trials have evaluated, in different settings, the efficacy of vaccine therapy against specific targets (4–6). To evade host immune surveillance, cancer cells can inhibit the immune system through inhibitory pathways such as cytotoxic T-lymphocyte-associated protein 4 (CTLA-4) or programmed-cell death 1 (PD-1) and its ligand (PD-L1). The activation of these pathways blocks the immune response of tumor-infiltrating lymphocytes, allowing the proliferation of tumor cells (7). Thus immunotherapy targeting PD-1 or PD-L1 has emerged as a new therapeutic strategy for NSCLC. Since 2015, 3 immune checkpoint inhibitors targeting PD-1/PD-L1, nivolumab, pembrolizumab, and atezolizumab, were approved for second-line treatment of advanced NSCLC (8–11). Since 2016, pembrolizumab, atezolizumab and cemiplimab also received approval in the first-line setting as a single agent for patients whose tumors have high PD-L1 expression (tumor proportion score TPS ≥ 50%) (12–15). The efficacy of immunotherapy in combination with platinum-based CT was also demonstrated in several large phase III randomized trials in patients with metastatic NSCLC, regardless of PD-L1 expression level (16–19).

Indoleamine 2,3-dioxygenase (IDO-1) is a cytosolic haeme-containing enzyme encoded by the *INDO* gene on human chromosome 8p22. It is expressed in various tissue and cell types, but the small intestine, epididymis, lung, female genital tract and placenta have been reported as its main sites of expression. The principal effect of IDO-1 is the intracellular catabolism of the essential aminoacid tryptophan to N-formil-kynurenine and its downstream metabolities (20). The IDO-1 activity of mouse placenta has been demonstrated to have an essential role in preventing rejection of allogenic fetuses (21). IDO-1 effects on immune suppression are due to decreased tryptophan availability and the generation of tryptophan metabolites, culminating in multipronged negative effects on T lymphocytes in proximity to IDO-1-expressing cells. T lymphocytes are extremely sensitive to tryptophan shortage, which causes their arrest in the G1 phase of the cell cycle (22). This deficiency can lead to “death by starvation” by inducing an accumulation of uncharged tryptophan-tRNA, which is sensed by the stress-response kinase GCN2, with the consequent prevention of T-cell activation (23). Some tryptophan metabolites also have the potential to induce apoptosis in lymphocytes. Moreover, the principal tryptophan metabolite kynurenine induces reversible T-cell anergy and seems to favor a regulatory phenotype in CD4^+^ T-cells (24–26). IDO-1 expression occurs in tumor-draining lymph nodes, in the peri-tumoral stroma and in tumor tissue as well. Accumulation of IDO-1-positive dendritic cells (DCs) was found in draining nodes from patients with melanoma, breast, colon, lung, pancreatic cancers and hepatocarcinoma (27). IDO-1 expression by tumor cells can be part of genetic changes involved in malignant transformation such as loss of Bin-1. Alternatively, IDO-1 in tumor cells could also be induced by Gamma-Interferon (IFN-γ) or other inflammatory mediators (28). IDO-1 expression and activity have usually been associated with a worse prognosis in ovarian carcinoma, endometrial carcinoma, osteosarcoma and colon carcinoma (29). Although in most studies IDO-1 expression has been associated with a worse outcome in several cancer types, some studies also reported positive prognostic effects (30). Currently, several clinical phase I and II trials with IDO-1-inhibitors in human cancer are reporting results. In most of these trials, IDO-1-inhibitors are administered in combination with either CT or other immunotherapeutic strategies targeting PD-1 and PD-L1. Data in metastatic tissue and blood have suggested that the expression of IDO-1 and other well-known immune checkpoint such as CTLA-4 and PD-L1 may be significantly interconnected (31–33); consequently, new possible combination therapies in lung cancer with IDO-1 and PD-1/PD-L1/CTLA-4 inhibitors may be explored. Combination therapies blocking several of these molecules simultaneously may be of particular interest.

Despite the increasing interest reported in the literature about IDO-1 and its correlation with immune suppression in cancer, scant information exists up to now regarding the expression of IDO-1 in lung cancer. In this study we investigated the expression of IDO-1 in two different cohorts of surgically-resected specimens of NSCLC, both in tumor cells and in tumor immune infiltrate, with specific correlation analyses of IDO-1 expression levels and clinical features of patients. We also evaluated the prognostic role of IDO-1 expression in terms of OS in different NSCLC sub-populations and its possible correlations with other immune checkpoints in this setting. With our analysis, based on IDO-1 expression, we strongly encourage to consider a possible IDO-1 inhibition therapy IDO-1 expression-driven and this could be the way to overcome the resistance observed in melanoma patients treated with IDO-1 inhibitors.

## Materials and methods

### Patients

For the IDO-1 expression analysis we used formalin-fixed paraffin-embedded (FFPE) tumor samples obtained from patients who underwent surgical lung resections at two different institutions. The principal inclusion criteria were histopathological diagnosis of NSCLC after complete surgery (lobectomy or pneumonectomy); exclusion criteria were sub-lobar resections and previous treatment with radiotherapy or chemotherapy. Clinical data and biologic variables of patients were collected: age, sex, smoking history, histology, grading and disease stage.

The first cohort of patients was obtained from NSCLC surgical patients at the Department of Oncology and Radiotherapy, Medical University of Gdansk (Poland) from April 2008 to August 2010. An experienced thoracic histopathologist selected the areas from blocks to obtain triplicate 1 mm cores for preparation of tumor microarrays (TMAs). For this cohort we analyzed 10 TMAs with a total of 264 tumor specimens, corresponding to 124 patients. The second cohort was provided by the Olivia Newton – John Cancer and Wellness Centre of Melbourne (Australia) and comprised early stage NSCLC cases sequentially resected between 1992 and 2010. An experienced thoracic histopathologist selected the areas from blocks to obtain triplicate 1 mm cores for preparation of TMAs. For this cohort, the analysis was performed on 20 TMAs, with a total of 1.165 tumor specimens, corresponding to 444 patients.

The surgical histology reports were reviewed and the lymph node and lung cancer stages were categorized by the eighth edition of the International Association for the Study of Lung Cancer (IASLC) TNM staging system. Prior to the use of clinical materials for investigation, informed consent from patients was obtained.

We used the first, smaller cohort to make an exploratory analysis; on the basis of the positive results obtained, without statistically significance because of the small number of patients, the second, larger cohort was used to validate previous results.

### Procedures

The immunohistochemistry (IHC) assessment was performed at one centralized laboratory (University of Colorado, Hirsch Biomarker Analysis Laboratory, CLIA #06D2003207 RRID number: SCR_004662). FFPE tumor samples (4 micron on charged glass slides) were stained using the Benchmark XT autostainer platform (Ventana Medical Systems/Roche, Tucson, AZ). Following addition of a bar-coded label, slides were baked at 60° for 1 hour. Slides were then treated with Cell Conditioning 1 (CC1) reagent for 60 minutes for antigen retrieval followed by staining with the IDO-1 primary rabbit monoclonal antibody D5J4E (#86630, Cell Signaling, Danvers, MA) diluted 1:200 with SignalStain antibody diluent (#8112) at RT for 1 hour. The ultraView DAB detection kit (#760-500, Roche) was used and slides were counterstained with Hematoxylin II for 4 minutes and post-counterstained with Bluing agent for 4 minutes. Following staining, slides were rinsed with detergent and water, then cleared and dehydrated on an automated Tissue-Tek Prisma platform (Sakura, Torrance, CA) and cover slipped using a Tissue-Tek Film cover slipper.

The evaluation and distinction between cancer and immune cells was performed by microscope during IHC scoring. Two separate scoring systems were employed, one for IDO-1 expression in tumor cells and one for IDO-1 expression in tumor immune infiltrate. Staining of tumor cells was scored in four different categories, including no staining (0), weak staining (1+, light brown membrane staining, visible only with high magnification), intermediate staining (2+, between 1+ and 3+) and strong staining (3+, dark-brown linear membrane staining, visible just with low magnification). The H-Score system was used to generate a semi-quantitative score, ranging from 0 to 300 and was calculated with the following formula: 1 x (percentage of 1+ cells) + 2 x (percentage of 2+ cells) + 3 x (percentage of 3+ cells). Staining of immune cells was scored in two different categories, including no staining (0) and positive staining (any dark-brown linear membrane staining, visible just with low magnification). The scoring was performed according to the percentage of immune cells with positive staining compared to total immune cells present in the tumor sample. The percentage of tumor and immune cells showing the different staining intensities was assessed visually by two independent pathologists (H.Y. and M.K.). The analysis was performed blinded, such that pathologists performing IHC evaluation were unaware of the other’s scoring.

**Figure 1:**
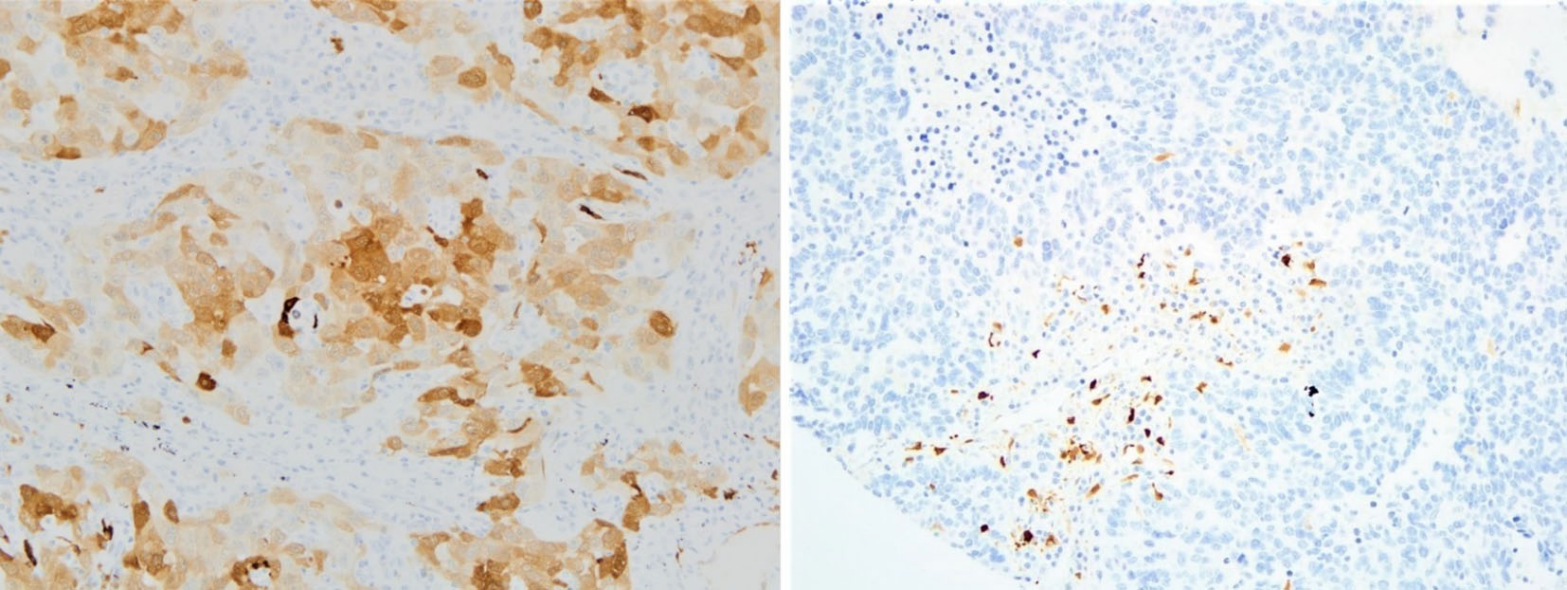
Examples of IDO-1 IHC positive tumor samples in tumor cells (on the left) and in the stroma - immune cells (on the right); both pictures are in 20x magnification (IDO-1, indoleamine 2,3-dioxygenase; IHC, immunohistochemistry).

IDO-1 expression by IHC status was considered independently in tumor cells and immune cells; it was considered as expressed in tumor cells with H-Score ≥ 1 and as expressed in immune cells with percentage ≥ 1% (**Figure 1**).

We consider IDO-1 expression in immune cells overall in the tumor microenvironment, though immune cells should not be considered as a single population, but represented by different subpopulations (macrophage, lymphocytes, stromal cells…). We observed that IDO-1 expression in tumor infiltrate was much higher as compared to tumor cells, but we did not perform scoring on each single subpopulation in order not to lose power and statistical significance.

### Statistical analysis

The IDO-1 H-Score was assessed for a cutpoint to identify those that are IDO-1-positive or negative (IDO-1+/-) by dichotomizing H-Score using the log-rank test to find a threshold that best discriminated survival times. A cutpoint of 1 resulted in the best separation at survival times. Therefore, a H-Score ≥ 1 was used to identify individuals as IDO-1+ and < 1 as IDO-1-. Correlation results comparing IDO-1 H-Score with demographic and biological variables were assessed using Spearman and Kendall tests; a value of 1 or -1 indicates perfect correlation, while a value of 0 indicates no correlation. A Cox proportional hazards (PH) model was used to assess if OS was associated with IDO-1 positivity, age, sex, stage, PD-L1 status (tumor proportion score TPS cutpoint of 5%), histology, percent positive IDO-1 immune cells and percent immune cells to tumor cells. Individuals with stage 4 disease were removed from the analysis due to the small numbers in this group (N = 7 [1.6%]); Univariate analyses for these variables were conducted. If a variable was found to be significant in the univariate setting, it was included in the multivariable analysis.

Similar analyses were conducted assessing the percent of immune cells positive for IDO-1. A cutpoint of 20% was found to be the threshold that best discriminated survival times using the log-rank test. Correlation results comparing IDO-1+ % immune cells with demographic and biological variables were also assessed with Spearman and Kendall tests. A Cox proportional hazards (PH) model was used to assess if OS was associated with IDO-1+ % immune cells, age, sex, stage, PDL1 status (tumor proportion score TPS cutpoint of 5%), and histology.

## Results

**Figure 2:**
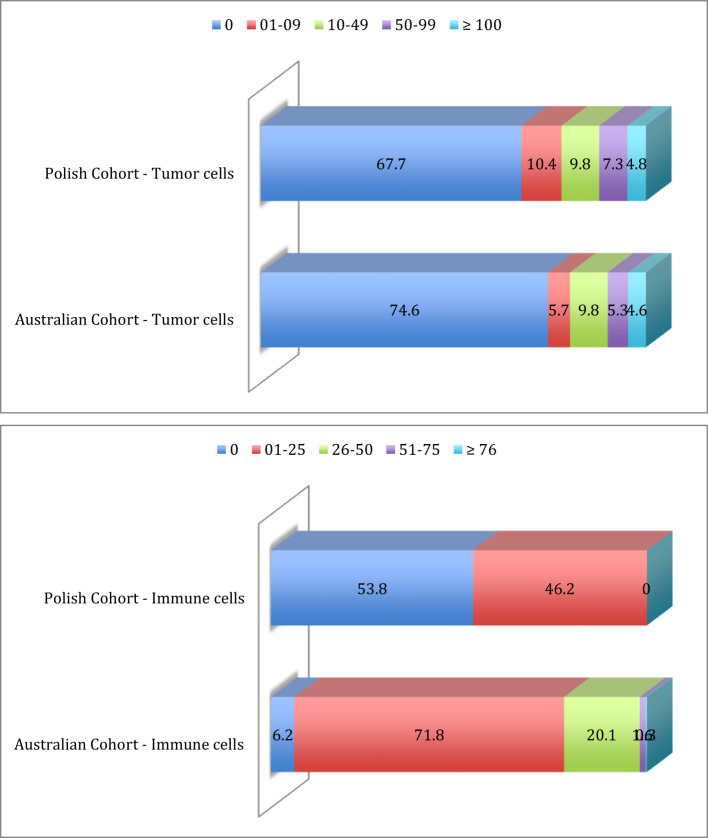
Prevalence distribution of IDO-1 expression in tumor cells and immune cells in Polish cohort and Australian cohort.

Exploratory analysis was performed on the Polish cohort of 124 patients in which 2 cases were excluded due to a lack of clinical data (see **Table 1**). Of the 122 remaining patients, corresponding to 259 evaluable tumor specimens, we analyzed IDO-1-expression by IHC in tumor cells, resulting in 84 patients (67.7%) IHC-negative (IHC-) and 40 patients (32.3%) IHC-positive (IHC+). Of the positive cases, 13 patients (10.4%) demonstrated a H-Score between 1 and 9, 12 patients (9.8%) had a H-Score between 10 and 49, 9 patients (7.3%) had a H-Score between 50 and 99 and 6 patients (4.8%) had a H-Score of more than 100 (**Figure 2**).

**Table 1:** Polish cohort patient characteristics according to IDO-1 IHC-status of tumor cells and immune cells.

	Overall population (N = 122)	Tumor cells (N = 122)		Immune cells (N = 117)	
		IHC+ (N = 40)	IHC- (N = 82)	IHC+ (N = 54)	IHC- (N = 63)
Age					
Median (range)	64.01 (37-77)	66.25 (37-76)	60.68 (45-77)	64.33 (37-77)	61.16 (45-77)
< 65 years	69 (56.6%)	17 (42.5%)	52 (63.4%)	30 (55.6)	38 (60.3)
≥ 65 years	53 (43.4%)	23 (57.5%)	30 (36.6%)	24 (44.4)	25 (39.7)
Sex					
Men	99 (81.1%)	31 (77.5%)	68 (82.9%)	44 (81.5)	51 (80.9)
Women	23 (18.9%)	9 (22.5%)	14 (17.1%)	10 (18.5)	12 (19.1)
Smoking					
Never	3 (2.5%)	2 (5.0%)	1 (1.2%)	3 (5.6)	0 (0.0)
Ever	119 (97.5%)	38 (95.0%)	81 (98.8%)	51 (94.4)	63 (100.0)
Histology					
ADC	35 (28.7%)	11 (27.5%)	24 (29.3%)	15 (27.8)	18 (28.6)
SCC	72 (59.0%)	26 (65.0%)	46 (56.1%)	32 (59.2)	35 (55.6)
LCC	4 (3.3%)	2 (5.0%)	2 (2.4%)	2 (3.7)	2 (3.2)
Others	11 (9.0%)	1 (2.5%)	10 (12.2%)	5 (9.3)	8 (12.6)
Staging					
I	44 (36.1%)	17 (42.5%)	27 (32.9%)	23 (42.6)	21 (33.2)
II	34 (27.9%)	12 (30.0%)	22 (26.9%)	16 (29.6)	18 (28.6)
III	35 (28.7%)	10 (25.0%)	25 (30.5%)	12 (22.2)	18 (28.6)
IV	7 (5.7%)	1 (2.5%)	6 (7.3%)	3 (5.6)	4 (6.4)
Unknown	2 (1.6%)	0 (0.0%)	2 (2.4%)	0 (0.0)	2 (3.2)
Grading					
G1	14 (11.5%)	3 (7.5%)	11 (13.4%)	5 (9.3)	8 (12.7)
G2	53 (43.4%)	17 (42.5%)	36 (43.9%)	24 (44.4)	25 (39.7)
G3	39 (32.0%)	16 (40.0%)	23 (28.0%)	15 (27.8)	25 (39.7)
Unknown	16 (13.1%)	4 (10.0%)	12 (14.7%)	10 (18.5)	5 (7.9)

IDO-1, indoleamine 2,3-dioxygenase; IHC, immunohistochemistry; N, number of patients; ADC, adenocarcinoma; SCC, squamous cell carcinoma; LCC, large cell carcinoma.

For 5 patients, IHC-staining in immune cells was not clearly detectable; of the 117 remaining patients, corresponding to 248 evaluable tumor specimens, tumor immune infiltrate was IDO-1 negative in 63 patients (53.8%), while in 54 patients (46.2%) tumor immune infiltrate was IDO-1 positive, in particular with a percentage of IDO-1+ immune cell between 1 and 25; there were no patients with a percentage higher than 25% (**Figure 2**).

Prevalence of IDO-1-expression (32.3% in the overall population) was higher in specific sub-populations of patients, in particular with age ≥ 65 years old (43.4%), female gender (39.1%), never smokers (66.6%), squamous histology (36.1%), stage I disease (38.6%) and poorly differentiated disease (41.0%). Survival analysis did not reach a statistically significant difference between patients with tumor IDO-1-expression (H-Score ≥ 1) and patients without. OS was 36.4 *vs* 33.3 months for patients with and without IDO-1-expression respectively (HR: 0.73, 95% CI 0.43-1.24, p = 0.27) and PFS was 27.8 *vs* 16.8 months for patients with and without IDO-1-expression respectively (HR: 0.76, 95% CI 0.46-1.25, p = 0.30). The OS benefit seemed to be higher when we applied an alternative cut-off, with H-Score ≥ 10; in this case OS HR was 0.69 (95% CI 0.38-1.25, p = 0.27). We identified some sub-populations with higher survival benefit derived from IDO-1-expression in tumor cells: in particular, for patients with stage I disease OS HR was 0.27 (95% CI 0.09-0.81, p = 0.07), PFS HR was 0.35 (95% CI 0.12-0.97, p = 0.09); for patients with adenocarcinoma OS HR was 0.37 (95% CI 0.12-0.79, p = 0.003), PFS HR was 0.21 (95% CI 0.08-0.58, p = 0.023).

Most results were not statistically significant because of the small number of patients included in this cohort. Nevertheless, based on the encouraging indications derived from these data, we used a second larger cohort in order to provide a higher number of patients and statistically validate previous results.

Thus, a second validating analysis was performed on the Australian cohort of 444 patients; 7 which lacked clinical records. Of these 437 patients, corresponding to 1,165 tumor specimens, we evaluated IDO-1-expression levels by IHC both in tumor cells and in immune infiltrate.

**Table 2** identifies the prevalence of IDO-1-expression in tumor cells was distributed as follow: 326 patients (74.6%) were IHC- and 111 (25.4%) were IHC+. For IHC positive cases, 25 patients (22.5%) had a H-Score between 1 and 9, 43 patients (38.8%) had a H-Score between 10 and 49, 23 patients (20.7%) had a H-Score between 50 and 99 and 20 (18.0%) patients had a H-Score of more than 100 (**Figure 2**).

**Table 2:** Australian cohort patient characteristics according to IDO-1 IHC-status of tumor cells and immune cells.

	Overall population (N = 437)	Tumor cells (N = 437)		Immune cells (N = 433)	
		IHC+ (N = 111)	IHC- (N = 326)	IHC+ (N = 406)	IHC- (N = 27)
Age					
Median (range)	67.3 (29.3-85.7)	68.4 (32.3-85.1)	67.3 (29.3-85.7)	67.4 (29.3-85.7)	65.9 (34.5-83.5)
< 65 years	181 (41.4%)	41 (36.9%)	140 (42.9%)	164 (40.4%)	13 (48.1%)
≥ 65 years	256 (58.6%)	70 (63.1%)	186 (57.1%)	242 (59.6%)	14 (51.9%)
Sex					
Men	301 (68.9%)	73 (65.8%)	228 (69.9%)	278 (68.5%)	21 (77.8%)
Women	136 (31.1%)	38 (34.2%)	98 (30.1%)	128 (31.5%)	6 (22.2%)
Smoking					
Never	31 (7.1%)	10 (9.0%)	21 (6.4%)	30 (7.4%)	1 (3.7%)
Ever	388 (88.8%)	95 (85.6%)	293 (89.9%)	358 (88.2%)	26 (96.3%)
< 20 packs/year	35 (9.0%)	6 (6.3%)	29 (9.1%)	31 (8.7%)	3 (11.5%)
≥ 20 packs/year	353 (91.0%)	89 (93.7%)	264 (80.9%)	327 (91.3%)	23 (88.5%)
Unknown	18 (4.1%)	6 (5.4%)	12 (3.7%)	18 (4.4%)	0 (0.0%)
Histology					
ADC	234 (53.5%)	64 (57.7%)	170 (52.1%)	225 (55.4%)	8 (29.6%)
SCC	153 (35.0%)	34 (30.6%)	119 (36.5%)	136 (33.5%)	15 (55.6%)
LCC	31 (7.1%)	9 (8.1%)	22 (6.8%)	29 (7.2%)	2 (7.4%)
Others	19 (4.4%)	4 (3.6%)	15 (4.6%)	16 (3.9%)	2 (7.4%)
Staging					
I	187 (42.8%)	54 (48.7%)	133 (40.8%)	177 (43.6%)	9 (33.3%)
II	130 (29.7%)	32 (28.8%)	98 (30.1%)	121 (29.8%)	7 (25.9%)
III	113 (25.9%)	23 (20.7%)	90 (27.6%)	101 (24.9%)	11 (40.8%)
IV	7 (1.6%)	2 (1.8%)	5 (1.5%)	7 (1.7%)	0 (0.0%)
EGFR status					
Wild-type	167 (38.2%)	43 (38.7%)	124 (38.0%)	155 (38.2%)	11 (40.7%)
Mutant	24 (5.5%)	8 (7.2%)	16 (4.9%)	22 (5.4%)	2 (7.4%)
Unknown	246 (56.3%)	60 (54.1%)	186 (57.1%)	229 (56.4%)	14 (51.9%)
KRAS status					
Wild-type	166 (38.0%)	43 (38.7%)	123 (37.7%)	154 (37.9%)	11 (40.7%)
Mutant	78 (17.8%)	17 (15.3%)	61 (18.7%)	73 (18.0%)	4 (14.8%)
Unknown	193 (44.2%)	51 (46.0%)	142 (43.6%)	179 (44.1%)	12 (44.5%)
PD-L1 status					
Negative	289 (66.1%)	65 (58.6%)	224 (68.7%)	264 (65.0%)	22 (81.5%)
Positive	129 (29.5%)	41 (36.9%)	88 (27.0%)	126 (31.0%)	3 (11.1%)
TPS ≥ 1%	16 (12.4%)	3 (7.3%)	13 (14.8%)	16 (12.7%)	0 (0.0%)
TPS ≥ 5%	15 (11.6%)	3 (7.3%)	12 (13.6%)	14 (11.1%)	1 (33.3%)
TPS ≥ 15%	12 (9.3%)	4 (9.8%)	8 (9.1%)	12 (9.5%)	0 (0.0%)
TPS ≥ 50%	86 (66.7%)	31 (75.6%)	55 (62.5%)	84 (66.7%)	2 (66.7%)
Unknown	19 (4.4%)	5 (4.5%)	14 (4.3%)	16 (4.0%)	2 (7.4%)

IDO-1, indoleamine 2,3-dioxygenase; IHC, immunohistochemistry; N, number of patients; ADC, adenocarcinoma; SCC, squamous cell carcinoma; LCC, large cell carcinoma; TPS, tumor proportion score.

IDO-1-expression in tumor infiltrate was much higher (as compared to tumor cells); for 4 patients IHC-staining was not clearly detectable and of the 433 remaining patients, tumor immune infiltrate was IDO-1- for just 27 patients (6.2%), while 406 patients (93.8%) were IDO-1+. For IHC positive cases, 311 patients (76.6%) presented a percentage of IDO-1+ immune cell between 1 and 25, 87 patients (21.4%) between 26 and 50, 7 patients (1.7%) between 51 and 75 and just one patient (0.3%) with percentage ≥ 76 (**Figure 2**).

Two separate scoring systems were realized: the H-Score system was used in tumor cells to generate a semi-quantitative score, ranging from 0 to 300; the scoring in immune cells was performed according to the percentage of immune cells with positive staining compared to total immune cells present in the tumor sample, ranging from 0% to 100%. IDO-1 expression was evaluated by immunohistochemistry on the Ventana Benchmark XT autostainer using a rabbit monoclonal antibody. IDO-1 expression by IHC status was considered independently in tumor cells and immune cells; it was considered as expressed in tumor cells with H-Score ≥ 1 and as expressed in immune cells with percentage ≥ 1%.

There was a significant positive correlation between IDO-1 positive tumor cells and immune cells (0.2167, p < 0.001). Both continuous and binary versions of tumor H-Score showed a significant positive correlation with the amount of tumor immune infiltrate (0.1806 and 0.1698, p < 0.0001, respectively).

**Figure 3:**
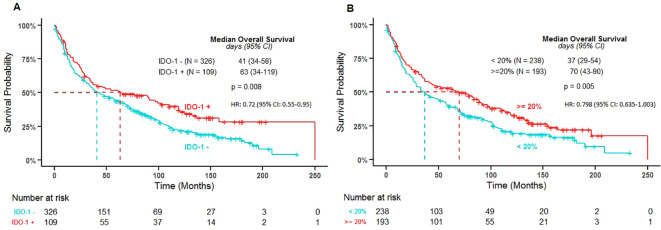
Overall survival by IDO-1 positivity in tumor cells **(A)** and in immune cells **(B)**. IDO-1 +/- and IDO-1% immune cells cut-points were determined by dichotomizing H-Score and the percentage of immune cells using the log-rank test to find optimal thresholds that discriminated survival times; these were found to occur at an H-Score of 1 for tumor cells and at 20% for immune cells. The P-value represents the log-rank test results from the Kaplan Meier estimates used to find the optimal thresholds that discriminated survival times for IDO-1 +/- and IDO-1%. (IDO: indoleamine 2,3-dioxygenase; N: number of patients; HR: hazard ratio from the multivariable Cox PH model IDO-1 positive *versus* IDO-1 negative and ≥ 20% *versus* < 20% respectively).

None of the analyzed variables (age, sex, histology, stage, EGFR, KRAS) were found to display a significant correlation with IDO-1 positivity in tumor and immune cells. IDO-1+/- was found to be significantly associated with OS in the univariate setting (P-value = 0.009) and remained significant in the multivariable model (P-value = 0.021). Those that were positive for IDO-1 were at significantly lower risk than those that were negative (HR: 0.72 [95% CI: 0.55-0.95]) (**Figure 3**). Also PD-L1 status was not significantly correlated with IDO-1-positivity both in tumor and immune cells.

**Figure 4:**
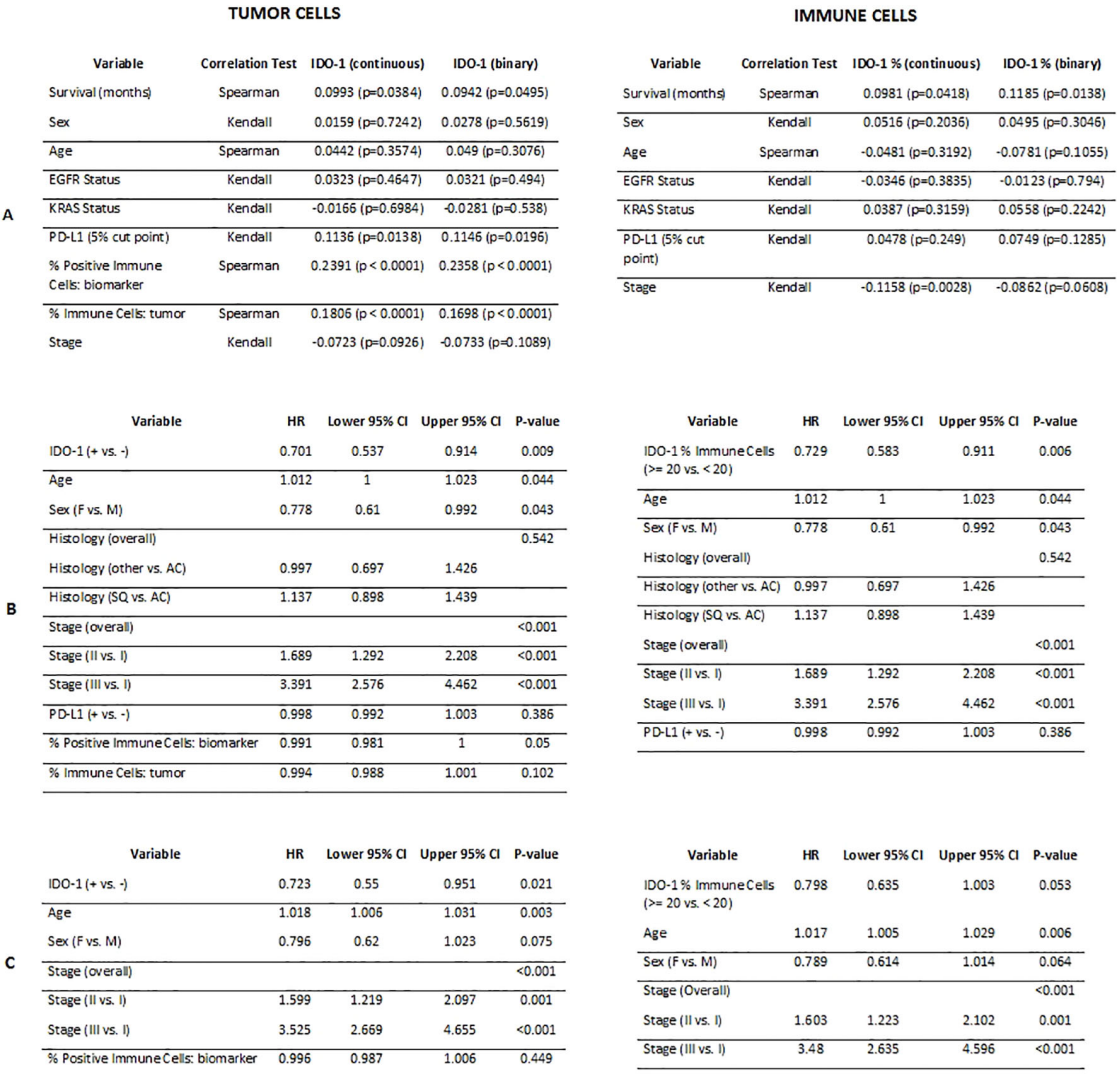
Correlation results comparing IDO-1 positivity in tumor cells and in immune cells (continuous and binary versions) with demographic and biological variables **(A)**; Cox PH univariate analysis results **(B)**; Cox PH multivariable analysis results **(C)**.

IDO-1+ % immune cells was found to be significantly associated with OS in the univariate setting (P-value = 0.006) and was borderline significant in the multivariable model (P-value = 0.053). Those with a percentage of immune cells positive for IDO-1 greater than 20% were at lower risk than those with less than 20% IDO-1+ immune cells (HR: 0.798 [95% CI: 0.635-1.003]) (**Figure 4**).

Age and stage were the only other variables that maintained significant associations with survival in the multivariable setting. In general, increased age and stage were related to worse OS.

## Discussion

The tryptophan catabolism enzyme IDO-1 has emerged as an intriguing target implicated in tumor immune escape. It’s now clear that to “get on the gas” of immune activation against tumors it is necessary to “get off the brakes” of tumor-associated immune suppression (34). One such braking mechanism receiving increasing interest involves IDO-1 expression.

In this study we evaluated the expression of IDO-1 in NSCLC, both in tumor cells and in tumor immune infiltrate, the prognostic role of IDO-1 expression in terms of OS in different NSCLC sub-populations and its possible correlations between other immune checkpoints in this setting. To our knowledge, this is the most extensive analysis of IDO-1 expression in NSCLC patients reported in the literature.

Our analysis showed that IDO-1 expression was low in tumor cells but much higher in the stroma and immune infiltrate. There was a significant correlation between IDO-1 positive tumor cells and immune cells; tumor H-Score also showed a significant positive correlation with the amount of tumor immune infiltrate. None of the analyzed variables (age, sex, histology, stage, EGFR, KRAS and PD-L1 status) were found to display a significant correlation with IDO-1 positivity in tumor and immune cells. IDO-1 positivity in tumor cells was found to be significantly associated with OS in the univariate setting and in the multivariable model where variables including age, sex, histology, stage, EGFR, KRAS and PD-L1 status were included. IDO-1 positivity in immune cells was found to be significantly associated with OS in the univariate setting and was borderline significant in the multivariable model. Age and stage were the only variables that maintained significant associations with survival in the multivariable setting. In general, increased age and stage were related to worse OS. The reason because other variables did not show any association in multivariable setting is unclear: a possible explanation could be the relatively small number of IDO-1 strong positive tumors. Evaluation of IDO-1 expression was performed in our study by IHC. A validated tool to evaluate the IDO-1 gene expression is also Real Time Polymerase Chain Reaction (RT-PCR) (35, 36). The choice to use IHC instead of RT-PCR was determined by lower costs, faster execution time on such a large number of specimens and higher reproducibility in the real clinical practice. Serum concentrations of tryptophan (Trp), kynurenine (Kyn) and Kyn/Trp ratio has been used as a surrogate of IDO-1 expression and several studies have shown that the Kyn/Trp ratio increases in the serum or plasma of patients with cancers, suggesting that enhanced IDO-1 activity may play a role in immunesuppression observed in cancer patients (37). Part of the inconsistencies in reported data on IDO-1 expression in tumor tissues and also in normal tissues may be due to this technical difference, such as the variability of commercial antibodies (monoclonal *versus* polyclonal, mouse *versus* rabbit *versus* goat), specific staining protocol and automated staining platform.

IDO-1 expression and activity has been reported in several human cancers, but there is a strong contradiction in the literature about its prognostic role; although in most studies IDO-1 expression has been associated with worse outcomes, some studies also reported a positive prognostic effect (30). This putative contradiction could be explained by different hypotheses, that support a possible positive role of IDO-1 in the tumor immune response: the IDO-1-induced tryptophane deprivation has been reported to also decrease tumor cell proliferation. Moreover, IDO-1 expression can be modulated by different cytokines, in particular by IFN-γ as well as other cytokines also can modulate IDO-1 expression (IFN-α, IFN-β, IL-10, IL-1, TGF-β) (38). During an anti-tumor immune response, large quantities of such pro-inflammatory cytokines are secreted and IDO-1 expression could be considered as a representative biomarker of an ongoing anti-tumoral immune activation.

The prognostic role of IDO-1 expression was evaluated also in patients with stage III NSCLC treated with fractionated radiotherapy (RT) (39). In particular, low baseline activity of IDO-1, evaluated with the Kyn/Trp ratio, was significantly associated with better survival as IDO-1 at baseline and post-RT correlated significantly with OS and PFS. Moreover, researchers have demonstrated that RT caused significant reductions in IDO-1 activity during therapy, but increased significantly post-RT, suggesting that IDO-1 activity may be suppressed by anti-tumor immunity in some patients as early as 2 weeks after starting RT. In addition, the Kyn/Trp ratio remains at low levels in the middle phase of RT (at 4 weeks), while increasing at later stages. Therefore, changes of these IDO-1-associated molecules during RT could be used as potential biomarkers to determine the optimal individualized RT schedule for each patient.

New compounds targeting IDO-1 have been under investigation in NSCLC and also in other malignancies such as melanoma, with no encouraging results. In particular, the ECHO-301/KEYNOTE-252 phase 3 trial in patients with unresectable or metastatic melanoma has failed to demonstrate an improvement of PFS and OS with the IDO-1 inhibitor epacadostat in combination with pembrolizumab compared with placebo. The usefulness of IDO-1 inhibition as a strategy to enhance anti-PD-1 therapy activity in cancer remains unclear. Blockade of IDO-1 using small molecule inhibitors in combination with immune checkpoint blockade induces prominent antitumor response in mouse models of brain tumors (40). IDO-1 inhibition studies did not include a selection by the IDO-1 expression.

It is unclear if the interaction between IDO-1, PD-L1 and a neoplastic microenvironment could have a significant correlation with patient outcomes or if they could be a potential double therapeutic target. In an immunohistochemistry study on a series of resected NSCLC patients conducted by Mandarano et al (41), authors found a strong relationship in 34 (17.62%) of cases presenting a co-expression of both high IDO-1 and PD-L1 (p = 0.0003). Literature studies on murine models and on human NSCLC have demonstrated that those two molecules are closely interconnected (42). These findings are not confirmed in our larger cohort analysis as PD-L1 status was not significantly correlated with IDO-1-positivity both in tumor and immune cells. PD-L1 and IDO-1 are differentially expressed in human lung carcinomas and have distinct staining patterns (31). PD-L1 has a predominant cytoplasmic/membranous distribution whereas IDO-1 expression has a predominant perinuclear staining pattern as expected for a cytosolic enzyme. Both PD-L1 and IDO-1 overexpression are induced by T_H_1/IFNγ signaling as occurs at the tumor-infiltrating lymphocytes (TILs) level and are significantly higher in the tumor compartment than in stromal cells. However, they have shown an exclusive pattern with infrequent co-expression as samples from lung carcinomas with prominent levels of one of the markers had typically low level of the other. The proportion of cases co-expressing PD-L1 and IDO-1 was 7.1% and 10.9% in the two different cohorts. These results suggest that most lung tumors use preferentially one immune evasion pathway, but controversial data in the literature require further investigations.

## Conclusion

In conclusion, immunotherapy represents the new standard of care for the treatment of advanced NSCLC, both alone or in combination with traditional chemotherapy. Several clinical trials are currently focused on possible immuno-modulation strategies and targeting IDO-1 could be considered one of them. In our analysis, IDO-1 protein expression was found to be significantly associated with OS in the univariate setting (P-value = 0.009) and remained significant in the multivariable model (P-value = 0.021). IDO-1 IHC+ % immune cells was found to be significant associated with OS in the univariate setting (P-value = 0.006) and was borderline significant in the multivariable model (P-value = 0.053). Despite this last limitation in our results, we could hypothesize the possible prognostic role of IDO-1 expression in tumor and immune cells, highlighting the relevance of IDO-1 detection in tumor tissue and the importance of further investigations in this setting.

## Data Availability

The original contributions presented in the study are included in the article/supplementary material. Further inquiries can be directed to the corresponding author.
